# Spatial tuning of translational optic flow responses in hawkmoths of varying body size

**DOI:** 10.1007/s00359-021-01530-1

**Published:** 2021-12-10

**Authors:** Rebecca Grittner, Emily Baird, Anna Stöckl

**Affiliations:** 1 Behavioral Physiology and Sociobiology (Zoology II), University of Würzburg, Würzburg, Germany; 2 Department of Zoology, Stockholm University, Stockholm, Sweden

**Keywords:** Vision, Optic flow, Flight control, Hawkmoth, Allometry

## Abstract

To safely navigate their environment, flying insects rely on visual cues, such as optic flow. Which cues insects can extract from their environment depends closely on the spatial and temporal response properties of their visual system. These in turn can vary between individuals that differ in body size. How optic flow-based flight control depends on the spatial structure of visual cues, and how this relationship scales with body size, has previously been investigated in insects with apposition compound eyes. Here, we characterised the visual flight control response limits and their relationship to body size in an insect with superposition compound eyes: the hummingbird hawkmoth *Macroglossum stellatarum*. We used the hawkmoths’ centring response in a flight tunnel as a readout for their reception of translational optic flow stimuli of different spatial frequencies. We show that their responses cut off at different spatial frequencies when translational optic flow was presented on either one, or both tunnel walls. Combined with differences in flight speed, this suggests that their flight control was primarily limited by their temporal rather than spatial resolution. We also observed strong individual differences in flight performance, but no correlation between the spatial response cutoffs and body or eye size.

**Supplementary Information:**

The online version contains supplementary material available at 10.1007/s00359-021-01530-1.

## Introduction

To safely navigate their environment, many flying animals rely on visual cues. Insects in particular obtain information about their own position, their flight speed, their course and distance to nearby objects from wide-field image motion generated as they move through the air (Srinivasan et al. 1999; Collett 2002; Egelhaaf et al. 2014) called optic flow (Koenderink 1986). The magnitude of the perceived translational optic flow (that is, optic flow generated by movement along, rather than rotation about, the animals’ body axes) reveals information about the structure of their surrounding environment (Schwegmann et al. 2014; Stürzl et al. 2016; Bigge et al. 2021). An environment with contrast edges perpendicular to the animals’ flight direction generates strong optic flow cues that can guide a number of flight control behaviours. Keeping a safe distance from potential obstacles is achieved with the so-called centring response, which balances the perceived front-to-back translational optic flow experienced in the lateral field of view of each eye, and thus enables them to maintain an equal distance between obstacles on each side. Insects compensate for imbalances in this lateral optic flow, and thereby minimise the risk of colliding with nearby obstacles, by steering towards the side experiencing the lower magnitude of optic flow (Kirchner and Srinivasan 1989; Serres et al. 2008; Baird et al. 2010; Dyhr and Higgins 2010; Kern et al. 2012; Stöckl et al. 2019). Translational optic flow cues are also used to control flight speed (David 1982; Baird et al. 2005, 2010; Fry et al. 2009), and height above the ground (Kennedy and Marsh 1974; Kuenen and Baker 1982; Baird et al. 2006, 2021) and below confining structures (Portelli et al. 2011). Optic flow cues also provide information about changes in course, thus guiding straight flight paths (Linander et al. 2017; Bigge et al. 2021). Moreover, motion parallax created by translatory self-motion is an important source of depth information for flying insects (Schwegmann et al. 2014), and helps to maintain a safe distance to obstacles (Lecoeur et al. 2019) and to fly safely through gaps (Ravi et al. 2019).

Whether insects can make use of the visual cues present in their environment depends on the characteristics of their eyes, and of their nervous system that subsequently processes the visual information (Baird et al. 2020). The spatial sampling base of the eyes’ optics limits the absolute spatial resolution for motion detection (Borst and Egelhaaf 1989). Since this, in turn, is limited by the absolute size of the individual optical elements (Land 1997), spatial acuity often scales with absolute eye size (Kiltie 2000). Moreover, insect species with larger body sizes also tend to have larger eyes, which in turn tend to have higher spatial resolution (Rutowski 2000; Jander and Jander 2002; Streinzer et al. 2016). Moreover, the neural processing of the information sampled by the eyes, both in the spatial and temporal domain, sets a limit to the optic flow responses of the insects (Borst and Egelhaaf 1989). The spatial and temporal tuning of photoreceptors (Laughlin and Weckström 1993; Frederiksen et al. 2008; Gonzalez-Bellido et al. 2011; Stöckl et al. 2017c), and as a result of motion neurons that process optic flow, can differ greatly between species with different natural habitats and lifestyles (O’Carroll et al. 1996, 1997; Stöckl et al. 2017c). Together, optical and neural tuning define the species-specific spatial and temporal cutoffs of the optic flow-based flight responses.

Spatial and temporal tuning to optic flow can vary not only between species, but also between individuals within the same species, particularly when they vary in body size. Allometric scaling of the optical sampling base of the eye, the interommatidial angle, has been observed in the apposition compound eyes of bees (Spaethe 2003; Kapustjanskij 2007; Taylor et al. 2019) and the neural superposition apposition eyes of fruit flies (Currea et al. 2018). Smaller individuals have reduced spatial resolution, caused by larger interommatidial angles in their overall smaller eyes. The consequences for optic flow-based flight control vary in different insect species and eye types (Spaethe 2003; Dyhr and Higgins 2010; Chakravarthi et al. 2016; Currea et al. 2018): while the spatial resolution of bumblebees scaled with body size in a target detection task (Spaethe 2003), no correlation between body size and spatial acuity was observed with wide-field sinusoidal gratings in a choice task (Chakravarthi et al. 2016) and in flight tunnel experiments (Dyhr and Higgins 2010). In fruit flies, on the other hand, the coarser spatial resolution of the eyes of smaller individuals manifested in their responses in an optic flow-based flight control task (Currea et al. 2018), though the effect was outweighed by a reduction in the temporal resolution of small flies, which compensated for the decrease in contrast sensitivity in the eyes of smaller individuals.

To date, our understanding of how spatial acuity and allometric scaling affect optic flow-based flight control is limited to insects with apposition compound eyes and it is unclear how these effects translate to a different optical system—superposition compound eyes. In this eye type, hundreds of neighbouring facets focus light onto a single rhabdom, acting as a functional lens with a largely increased aperture compared to a single facet diameter (Exner 1891). Therefore, superposition compound eyes have a strongly increased sensitivity compared to apposition eyes (Snyder 1977; Warrant and McIntyre 1993; Land et al. 1997). This might result in different selection constraints on superposition compound eyes scaling with body size. For example, the need to sacrifice spatial and temporal resolution to preserve contrast sensitivity in small-eyed individuals observed in apposition compound eyes (Currea et al. 2018) might be reduced in superposition compound eyes. This would mean that individuals with smaller eyes may have the same minimum resolution as larger individuals.

Here, we investigate how allometric scaling affects the spatial limitations of flight control behaviour in an insect with superposition compound eyes, the hummingbird hawkmoth *Macroglossum stellatarum.* These insects are diurnal, allowing us to compare the scaling relationship to insects active in similar light environments (Spaethe 2003; Currea et al. 2018; Taylor et al. 2019). Moreover, these hawkmoths share habitats and food plants with the well-investigated bumblebee species *Bombus terrestris* (Stöckl and Kelber 2019), and therefore, extract their required optic flow information from similar visual environment. On the other hand, the hovering flight mode of the hummingbird hawkmoth and their phylogenetic distance to bees and flies also provide an interesting comparison for the role of spatial structure in optic flow-based flight control.

**Fig. 1:**
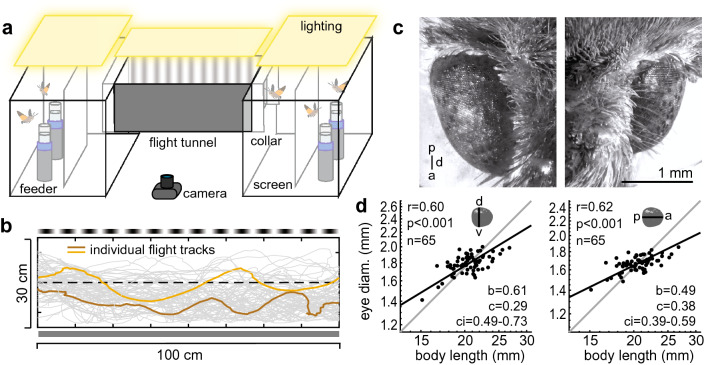
Experimental setup and study animals. **a** Our setup consisted of two flight cages (60 cm × 60 cm × 60 cm) connected by a flight tunnel (100 cm × 30 cm × 30 cm) through which the animals could cross freely. White screens in the middle of the two flight cages obstructed most visual cues from the flight cages during the tunnel crossing. At the tunnel entrances, white collars prevented unintended tunnel entrances and served as further visual shields. An opaque diffuser was placed on top of the tunnel to ensure uniform lighting and to remove any cues from the ceiling. The floor of the tunnel was covered with a semi-transparent diffuser so that the flight paths could be recorded from below. Different sinusoidal grating patterns were presented on the tunnel walls. **b** Examples of flight paths (two individual paths are highlighted in brown) in the asymmetric stimulus condition (one tunnel wall covered with 50% grey, the other covered with a sinusoidal grating). **c** Eyes of two individuals of *Macroglossum stellatarum* (body length: left = 26 mm, right = 16.8 mm), in dorsal view. **d** Relation between body length and eye diameter (d–v: measured from the dorsal to ventral side, a–p: measured from the anterior to posterior side of the eye). The strength of the linear correlation coefficient of the log-transformed data is given by r, and the statistical significance of the Pearson correlation coefficient by p. The allometric relationship derived from reduced major axis regression is given by the black line (exponential scaling exponent: b, normalisation constant: c, confidence interval of b: ci). The grey line represents isometric scaling

We used a behavioural approach similar to previous experiments performed on bumblebees (Dyhr and Higgins 2010; Chakravarthi et al. 2017, 2018) to assess the spatial acuity of hawkmoth flight control behaviour: a flight tunnel paradigm (Stöckl et al. 2019; Bigge et al. 2021), in which the centring response provides a readout for the reception of the translational optic flow stimuli of different spatial frequencies. We tested two stimulus configurations: symmetrical and asymmetrical optic flows (Fig. 1a, b). By testing individuals of a range of body and eye sizes (Fig. 1c, d), we determined whether there was an allometric scaling of the spatial acuity of hummingbird hawkmoths in this task. Interestingly, we found different spatial response cutoffs in two stimulus conditions, but no correlation between body or eye size and the flight control responses of the moths. Nonetheless, we could relate the different spatial cutoffs to the average speeds at which the animals crossed the tunnel in the two stimulus conditions, suggesting that the perception of optic flow is limited by temporal rather than spatial resolution.

## Materials and methods

### Animals

Male and female *Macroglossum stellatarum* (Sphingidae) were obtained from a colony in Würzburg, Germany, which was raised on their native host plant *Gallium* sp*.* The adult animals were kept on a 14:10 h light:dark cycle in flight cages (60 cm × 60 cm × 60 cm) and fed with artificial feeders (Pfaff and Kelber 2003) that contained a 20% sucrose–water solution. To investigate the effect of body and eye size on flight performance, the selected individuals had a wide range of body sizes (Fig. 1d). Animals used in the experiments were between 3 days and 2 weeks of age. To identify each hawkmoth individually, all animals were marked with a special code consisting of two different colours (Künstler-Acrylfarbe, Creabox Marabu) placed on the upper and lower abdomen after removing their scales at these positions (Fig. S1A). After each experiment, individuals were photographed using a digital camera (ELP USB-Camera, 2.0 Megapixel (1080p), Ailipu Technology) with an objective of 8 mm focal length to measure their total body length in Fiji (Schindelin et al. 2012) (Fig. S1A). We also measured eye size (the dorsal–ventral and posterior-anterior diameter) in Fiji (Fig. S1B), after photographing the eyes laterally using a Flexacam C1 (Leica) camera mounted on a M80 stereomicroscope with 10 × oculars and 1 × objective (Leica). Images were stitched using Picolay (www.picolay.de, Heribert Cypionka, Version: 2020-12-26).

### Experimental setup

The experiments were conducted using a flight tunnel (100 cm × 30 cm × 30 cm) with wooden walls and a Plexiglas floor and ceiling. The flight tunnel was connected to a flight cage (60 cm × 60 cm × 60 cm) on either side. The flight cages and the tunnel were lit from above with daylight-like fluorescent tubes (Osram L 18W/965 Biolux Tageslicht G13): two 1-m-long tubes lit the tunnel, and four 60-cm-long tubes lit each cage (Fig. 1a). All lights were connected to an electrical ballast (GloMat 2 × 40 W, Hagen), which increased the flicker frequency of the fluorescent tubes above 25 kHz, well outside of the resolvable range of the hawkmoths (Stöckl et al. 2019). The resulting light intensities in the tunnel were 1150 lx pointing up, 370 lx pointing down, 750 lx towards the exit of the tunnel and 670 lx towards the sides, measured from the centre of the tunnel (obtained with a lux meter (HT309 digital lux meter, HT Instruments). To minimise external visual cues, the ceiling of the tunnel was covered with a white felt blanket, which acted as a diffuser. The tunnel floor was covered with gauze to minimise reflections and visibility, while still making it possible to record the hawkmoths from below. Each flight cage contained two artificial feeders placed behind a white screen, which prevented the hawkmoths from seeing the feeders or other visual cues from the flight cage while crossing the tunnel. A camera (PS3-camera, Playstation eye, Sony) was placed below the tunnel to record each flight path at 50 Hz with image sizes of 640 × 480 pixels. The camera was controlled using ContaCam software (version 7.9.0 beta7, Contaware). The software’s motion detector was triggered by moths crossing the central 30 cm of the tunnel and saved a video that recorded 4 s before and after it was triggered. The camera recorded the central 90 cm of the tunnel (measured at 15 cm height). To assign a specific flight path to an individual hawkmoth, a camera (ELP USB-Camera, 2.0 Megapixel (1080p), Ailipu Technology) was placed above each tunnel entrance to record hawkmoths entering and exiting the tunnel using the ContaCam software. These videos were then analysed to identify the colour markers of each hawkmoth and thereby assign the recorded flight path to this individual.

Visual patterns were generated as printouts, which were subsequently laminated to reduce reflections. A uniform 50% grey stimulus was used as the control condition, and sinusoidal gratings of different wavelengths (Table 1) with 87% Michelson contrast were used for assessing the spatial response properties. The presented wavelengths had widths of 0.1, 0.2, 0.5, 0.8, 1.3, 1.6, 2.5, 3.3, 5, and 10 cm, resulting in spatial wavelengths of 2.62, 1.31, 0.52, 0.33, 0.20, 0.17, 0.11, 0.08, 0.05, and 0.03 cyc/deg as measured from the midline of the tunnel. In the *symmetric* configuration, the same stimuli were presented on both tunnel side walls, while in the *asymmetric* configuration, one tunnel side presented the uniform grey stimulus, and the other “variable” wall showed one of the sinusoidal grating patterns. In the control condition in both configurations, both tunnel side walls were grey.

**Table 1:** Pattern wavelengths and the corresponding spatial frequencies viewed at 90°–71.4° and 82.8° (median viewing angle for symmetric to asymmetric pattern changes and vice versa)—lateral to the tunnels’ midline

Wavelength (cm)	0.1	0.2	0.5	0.8	1.3	1.6	2.5	3.3	5	10
Spatial frequency at 90° (cyc/°)	2.62	1.31	0.52	0.33	0.20	0.16	0.10	0.08	0.05	0.03
Spatial frequency_s-a_ at 71.4° (cyc/°)	2.91	1.46	0.58	0.36	0.22	0.18	0.12	0.09	0.06	0.03
Spatial frequency_a-s_ at 82.8° (cyc/°)	2.66	1.33	0.53	0.33	0.20	0.17	0.11	0.08	0.05	0.03
Temporal frequency (Hz)—*asymmetric*	883.8		186.7		65.4	52.0	30.9	20.6	14.5	6.9
Temporal frequency (Hz)—*symmetric*		459.4	141.1	111.6	41.2	32.7	20.9	18.3	9.9	5.3

The temporal frequencies were calculated based on the hawkmoths’ average flight speed in each pattern wavelength condition. Cells without temporal frequency data were not tested in the respective conditions (symmetric/asymmetric)

To test the position in the visual field at which hawkmoths responded to the presented translational optic flow, we displayed a sinusoidal pattern of 2.5 cm wavelength on one tunnel wall, and the same pattern on the opposite wall, which changed to a uniform grey pattern halfway along the tunnel. We presented the wall with the change in pattern on both sides of the tunnel to control for side biases (Fig. 4a, b, respectively).

### Experimental procedure

After the hawkmoths were colour-marked, they were released into one of the flight cages connected to the tunnel. To ensure that the hawkmoths had enough time to explore the flight cage and the position of the feeders, the tunnel entrance was closed for the first half day. The tunnel was then opened for the second half of the day, and the hawkmoths were free to fly through and explore the second flight cage. To encourage exploration, we additionally moved moths between cages.

After the initial familiarisation with the setup, experiments were conducted with an open flight tunnel, which the hawkmoths could traverse freely. In all conditions, the wall patterns were presented in a pseudo-randomised order on consecutive days. Each pattern was tested for at least 6 h from 9:00 to 16:00 in the *symmetric* configuration, though the sampling would be repeated for an additional 6 h if too few flights were obtained. In the *asymmetric* conditions, patterns were swapped after the first 6 h for an additional 6 h to control for a possible side bias. Three different groups of hawkmoths (approx. 40 individuals each) were used to test the spatial responses in the *asymmetric* and *symmetric* configurations and the pattern switch experiment. The training and presentation order of the patterns was similar for all hawkmoths within each cohort.

We also conducted a smaller number of experiments with hawkmoths that flew through the flight tunnel individually in the *asymmetric* and *symmetric* configurations. They were separated into Plexiglas vials for the entire duration of the experiment, and released individually into one flight cage without a feeder, before being allowed to cross the tunnel to the opposite flight cage that contained a feeder. Before the experiment started, the hawkmoths were familiarised with the cage containing the feeder and allowed to feed briefly to increase their motivation. They were then moved to the opposite flight cage and allowed to warm up on a platform in front of the tunnel entrance. If they did not take off after 5 min, they were removed and tested the next day. As soon as a hawkmoth took off and crossed the tunnel, it was allowed to feed shortly at the feeders behind the screen before being caught and put back into the starting cage to cross the tunnel again. This procedure was repeated as often as the moths were motivated to fly through the tunnel. For this individual flight experiment, the setup was slightly modified: a collar was placed around the right tunnel entrance with gauze attached to it, forming a funnel to guide the hawkmoths into the tunnel. Since no qualitative difference between population and individual experiments was observed, the data from both were pooled in the analysis.

### Data analysis

We analysed the hawkmoths’ flight responses as previously described (Stöckl et al. 2019). In brief, the recorded videos were sorted to only include flights in which a single hawkmoth crossed the tunnel in one uninterrupted motion from one side to the other. Flights during which the hawkmoths did not cross the entire tunnel, crashed against one of the walls, or attempted to land on one of the walls, as well as cases in which more than one hawkmoth flew through the tunnel at the same time, were excluded. Further analysis was performed in Matlab (R2017a; The Mathworks). The flight path of each animal was automatically extracted from the video files using custom-written Matlab-software and, where this was not possible, semi-automatically tracked using the DLTdv6 software for Matlab (Hedrick 2008). The cameras recorded only the central 90 cm of the tunnel reliably, of which only the central 60 cm were analysed, to exclude the portions of flight tracks that might have been influenced by visual cues from the flight cages, and that often contained short phases of hovering when entering and exiting the tunnel (Stöckl et al. 2019). The median lateral position of each hawkmoth, the median speed (averaged from a frame-by-frame estimate) and the lateral movement (the relative amount of movement perpendicular to the tunnel direction, relative to the movement parallel to the tunnel averaged across frames) were extracted from each flight path. When the residuals of parametric tests were not normally distributed, the non-parametric Kruskal–Wallis test (with Bonferroni-corrected post hoc comparison) was used to compare the median position, lateral movement and average speed parameters across patterns with different spatial frequencies (Table S1, S2). For the *asymmetric* conditions, we used a Mann–Whitney *U* test to compare the median flight track positions to the midline position (0 cm), to assess whether the population of hawkmoths crossed at the midline of the tunnel. Furthermore, we used a Brown–Forsythe test to compare the variance of median positions in the *symmetric* conditions, as a measure for the strength of the centring response. The change in position and flight speed between flights from an *asymmetric* to a *symmetric* optic flow scenario in the pattern switch experiment was also assessed using the Kruskal–Wallis test.

To analyse individual effects on the three flight parameters (Figs. 5, 6), as well as their possible correlation with body length, we separated the patterns into resolvable and unresolvable wavelengths. This avoided pooling responses from visual conditions that induced stark changes in flight parameters, which might obscure potential size-related effects (larger moths flying faster, for example). Resolvable pattern wavelengths were defined as those that induced significant translational optic flow responses, and the finer patterns and the control condition for which no significant optic flow responses were observed were defined as unresolvable.

The resolvable patterns in the *symmetric* configuration included all wavelengths equal to and larger than 1.3 cm, and in the *asymmetric* configuration equal and larger than 2.5 cm. All smaller wavelengths in the respective configurations, and the grey condition, were analysed as the unresolvable group. To assess whether individual differences in flight parameters existed, we analysed the data in the resolvable and unresolvable groups using a Kruskal–Wallis test with individual ID as the grouping variable (results shown as *ID* in Figs. 4, 5). A Pearson correlation test was used to test for a linear correlation between individual flight performance and body length (results shown as *SC* in Figs. 4, 5).

We tested the fixed effects of body size (*large* or *small*, separated at the median body size of 20.52 mm for the *asymmetric*, and 20.28 mm for the *symmetric* configuration) and pattern condition (*resolvable* or *unresolvable*) on flight parameters using a linear mixed-effects model (lme4 in R v3.5.1) (Bates et al. 2015). We tested the significance of the fixed effects and their interaction for the model fit by comparing the Akaike information criterion (AIC) using a likelihood ratio test. To assess how the fixed effects impacted the flight parameters, we conducted post hoc comparisons with Tukey contrasts.

**Fig. 2:**
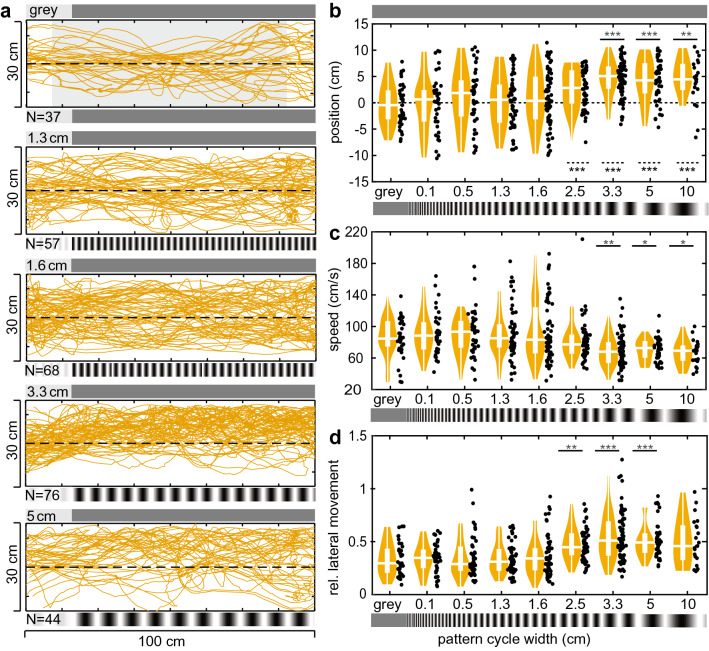
Flight responses of the hawkmoths in the *asymmetric* configuration. **a** Flight paths of each hawkmoth in the tunnel with patterns of different spatial frequencies (from top to bottom): control condition (grey on both walls), 1.3 cm, 1.6 cm, 3.3 cm, and 5 cm pattern cycle width on one wall and grey on the other. The number of analysed flights per pattern is indicated as *n*. **b** Median position, **c** average speed and **d** lateral movement of the flight paths in each condition. The number of trajectories N for each pattern condition shown was: grey *N* = 37, 0.1 *N* = 40, 0.5 *N* = 46, 1.3 *N* = 57, 1.6 *N* = 68, 2.5 *N* = 53, 3.3 *N* = 76, 5 *N* = 44, 10 *N* = 23. The results of the statistical comparison of the flight parameters in the different spatial conditions to the grey condition are indicated by black asterisks (Kruskal–Wallis test with Bonferroni-corrected post hoc comparison, Table S1). Red asterisks show the statistical result of testing the median position against the midline (Mann–Whitney *U* test, Table S1). *p < 0.05, **p < 0.01, ***p < 0.001. Boxplots (white) within the violin plots display the median, the 25th and 75th percentile and the whiskers denote the data range excluding outliers (values extending more than 1.5 interquartile ranges from the upper and lower box limits)

**Fig. 3:**
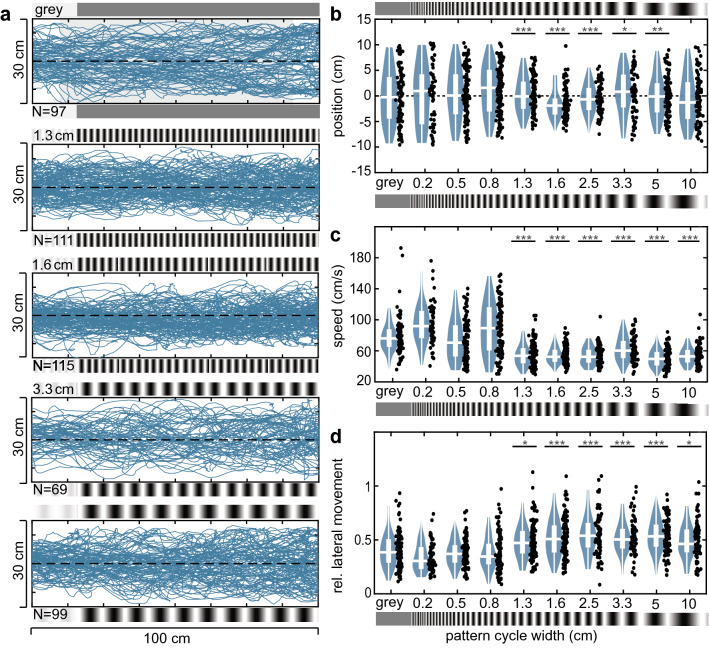
Flight responses of the hawkmoths in the *symmetric* configuration. **a** Flight paths of each hawkmoth in the tunnel with patterns of different spatial frequencies (from top to bottom): control condition (grey on both walls), 1.3 cm, 1.6 cm, 3.3 cm, and 5 cm pattern cycle width. The number of analysed flights per pattern is indicated as *n*. **b** Median position, **c** average speed and **d** lateral movement of the flight paths in each condition. The number of trajectories N for each pattern condition shown was: grey *N* = 97, 0.2 *N* = 59, 0.5 *N* = 107, 0.8 *N* = 114, 1.3 *N* = 111, 1.6 *N* = 115, 2.5 *N* = 78, 3.3 *N* = 69, 5 *N* = 99, 10 *N* = 81. Black asterisks in **b** show the results of a statistical comparison of the variance between the spatial conditions and the control group (Brown–Forsythe test, Table S2). Black asterisks in **c**, **d** show the results of statistical comparison of the different spatial conditions against the grey condition (Kruskal–Wallis test with Bonferroni-corrected post hoc comparison, Table S2). *p < 0.05, **p < 0.01, ***p < 0.001. Boxplots (white) within the violin plots display the median, the 25th and 75th percentile and the whiskers denote the data range excluding outliers (values extending more than 1.5 interquartile ranges from the upper and lower box limits)

We further analysed the hawkmoths’ responses for a correlation between spatial frequency cutoff and average eye diameter (Fig. 7), as well as body length (Fig. S3). We only analysed individuals that completed a flight in a high spatial wavelength condition (grey, 0.1, 0.2, 0.5, 0.8 cm), medium spatial wavelength condition (1.3, 1.6, and 2.5 cm) and low spatial wavelength condition (3.3, 5, 10 cm). The hawkmoths’ lateral movement was used as an indicator of their optic flow response (Fig. 7a, e), as it reliably correlated with the optic flow responses in the *asymmetric* (Fig. 2d) and *symmetric* configurations (Fig. 3d). For each individual, data from all available flight tracks were used to fit a normalised sigmoid response curve (Fig. 7b, f). Only hawkmoths for which a clear change in lateral movement between the highest and lowest spatial wavelength condition available were included in the analysis. As a measure for the spatial response cutoff, we used the 50% turning point of the sigmoid curves, and extracted the spatial wavelength at which it occurred. We furthermore calculated the spatial frequency perceived by the hawkmoths at 90° viewing angle, based on their median lateral position in the tunnel. We tested for a correlation between these spatial wavelength cutoff and eye diameter (Fig. 7c, g), as well as body length (Fig. S3A, E). To analyse whether the average speed of an individual affected their spatial response cutoff, we assessed a correlation between average speed and spatial wavelength cutoff (Fig. 7d, h). We furthermore calculated the perceived spatial frequency based on the median position in the tunnel, and checked for correlations with the individuals’ eye diameter (Fig. S3C, G), body length (Fig. S3B, F), and average flight speed (Fig. S3D, H). A Pearson correlation test was used to analyse a linear correlation between spatial cutoff frequency and body length, as well as between the spatial response cutoff and average flight speed.

## Results

We used a flight tunnel to assess the spatial tuning of the translational optic flow responses of hummingbird hawkmoths using sinusoidal grating stimuli of different wavelengths. Since their responses to translational optic-flow stimuli of different spatial frequencies have not been described in this paradigm before, we first tested the hawkmoths’ responses on a population level in two different stimulus configurations: an *asymmetric* and a *symmetric* configuration (Fig. 1a, b). Once the spatial response ranges were established, and suitable readouts for their flight control responses were determined, we assessed whether the body and eye size of the hawkmoths had an effect on their flight control responses to different spatial pattern wavelengths.

### Responses to asymmetric translational optic flow cues

We first studied the population responses of hawkmoths in the *asymmetric* configuration commonly used in bee flight-control experiments, which had sinusoidal patterns on one tunnel wall and a uniform grey pattern on the other. We expected the hawkmoths to increase their distance from the pattern while they were able to resolve it. If the spatial frequency was too fine to be resolved, we expected the flights to be similar to the control condition (grey on both walls) and equally distributed about the midline. Indeed, the median lateral positions of the flight tracks were significantly different from the control condition for spatial wavelengths of 3.3 cm and larger (Fig. 2a, b, Table S1), and significantly different from the midline for wavelengths of 2.5 cm and larger (Fig. 2a, b). Based on previous work (Stöckl et al. 2019; Bigge et al. 2021), we also expected a decrease in average flight speed when the hawkmoths were able to resolve the presented patterns, which was observed for spatial wavelengths equal or larger than 3.3 cm (Fig. 2c). At 2.5 cm, the reduction in average flight speed was not significantly different from the control condition. There was, however, a distinct decrease in flight speed variance at 2.5 cm, similar to the variance at larger pattern wavelengths, indicating that the 2.5 cm pattern might have been resolved by some individuals, though not enough to result in a statistically significant population effect. This notion was further confirmed by the analysis of the relative proportion of lateral movement contained in the hawkmoth’s flight tracks, caused by the avoidance of the patterned tunnel wall, which significantly increased for pattern wavelengths equal or larger than 2.5 cm (with the exception of the 10 cm pattern, Fig. 2d). Thus, the finest pattern wavelength at which the hawkmoths showed typical translational optic flow responses in the *asymmetric* configuration was 2.5 cm, which corresponds to 0.106 cyc/°as viewed at a 90° angle from the tunnel midline (Table 1).

### Responses to symmetric translational optic flow cues

In the *symmetric* configuration, patterns of the same spatial frequency were presented on both walls of the tunnel. Here, the centring response was used as an indicator for the response strength to the perceived translational optic flow. The hawkmoths’ flight paths increased in their concentration around the tunnel centre for wavelengths up to 1.6 cm but decreased again for coarser wavelengths (Fig. 3a). The centring strength, quantified as the variance in median positions, was significantly different from the control condition for patterns ranging from 1.3 to 5 cm (Fig. 3b, Table S2). In contrast to the relatively weak absolute reduction in flight speed in the *asymmetric* configuration, the average flight speed in the *symmetric* configuration decreased significantly to about 60% of the control condition for pattern wavelengths of 1.3 cm and larger (Fig. 3c). The spatial cutoff determined from the changes in average flight speed was the same as for the centring response. It was also mirrored in the lateral movements of the hawkmoths, which significantly increased for resolvable pattern wavelengths compared to the control condition or fine patterns (Fig. 3d). Thus, in the *symmetric* configuration, all flight parameters pointed to a spatial response limit at a wavelength of 1.3 cm, corresponding to 0.201 cyc/°as viewed 90° laterally in the hawkmoths’ field of view (Table 1).

### Average viewing angle for translational optic flow

**Fig. 4:**
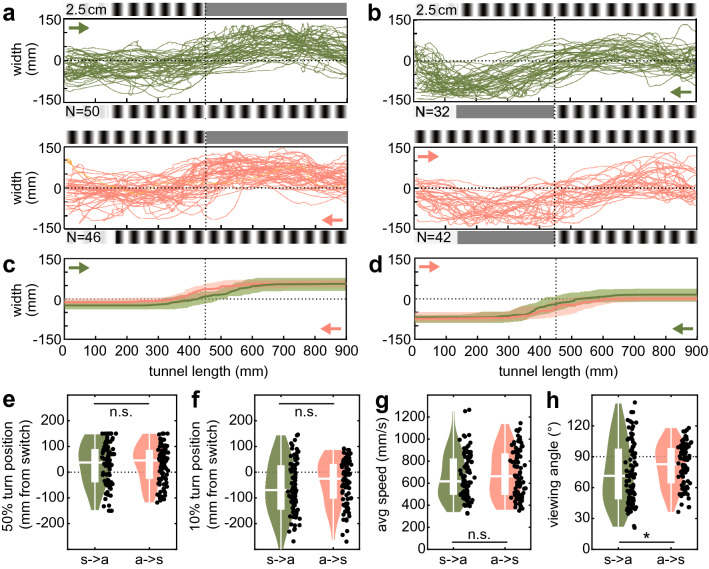
Response distance to a switch in translational optic flow. **a**, **b** Both tunnel walls presented a 2.5 cm wavelength pattern, which switched to a uniform grey pattern in the middle of one of the tunnel walls. To control for side biases, the pattern switch was presented on each of the two tunnel walls separately. Since the hawkmoths could enter the tunnel from both sides, this resulted in two flight conditions: flight from symmetric to asymmetric optic flow (*green*) and from asymmetric to symmetric (*red*). Each flight track was fitted with a sigmoid curve, the median and interquartile range of which is presented in **c** and **d**. **e** The 50% turning point of the sigmoidal fits and **f** the 10% turn point from baseline were compared for both flight groups, relative to the pattern switch position. **g** The average speed of the hawkmoths before they responded to the pattern change (in the first 300 mm of the tunnel) was compared between conditions. **h** depicts the perceived viewing angle of each individual hawkmoth, based on its 10% turn position and distance to the pattern-switch wall. The statistical comparisons in **e**, **g**, **h** were performed using a Kruskal–Wallis test (*Χ*^2^ = 0.0047, *p* = 0.94; *Χ*^2^ = 0.87, *p* = 0.35; *Χ*^2^ = 4.67, respectively, *p* = 0.03), **f** using an ANOVA (*F* = 2.6, *p* = 0.11). Boxplots (white) within the violin plots display the median, the 25th and 75th percentile and the whiskers denote the data range excluding outliers (values extending more than 1.5 interquartile ranges from the upper and lower box limits)

Our experiments so far yielded the spatial pattern wavelengths at which the hawkmoths stopped responding to the optic flow information presented on the tunnel walls. However, since the patterns were presented on tunnel walls parallel to the animals’ flight direction, the effective spatial wavelength of the patterns the hawkmoths perceived depended on which part of their receptive field they integrated the pattern information from. A pattern registered directly laterally, at 90° from their midline, stretches a wider angular width per cycle than the same pattern registered at 45° from the midline—and thus appears as a coarser spatial frequency than if the hawkmoth was “looking” at 45° frontally instead. To determine the effective spatial cutoff frequencies, we tested at which visual angle hawkmoths reacted to the wall patterns. We adopted an approach used in previous studies on bumblebees to determine this viewing angle (Baird et al. 2010; Linander et al. 2015). To do so, we displayed a sinusoidal pattern of 2.5 cm wavelength on one tunnel wall, and the same pattern on the opposite wall, which changed to a uniform grey pattern halfway through the tunnel (Fig. 4a, b). By analysing the position of the hawkmoths’ responses to this change in the perceived translational optic flow (Fig. 4c, d), we determined the minimum angle (measured from their midline) at which hawkmoths responded to the tunnel patterns.

We presented the wall with the change in pattern on both sides of the tunnel to control for side biases (Fig. 4a, b, respectively). Since the hawkmoths could enter the tunnel from both sides (Fig. 4, yellow and green tracks), this resulted in two stimulus conditions: flight from *symmetric* to *asymmetric* optic flow and vice versa, replicated in the two groups. We found that the hawkmoths adjusted their flight paths according to the perceived optic flow in all conditions. To quantify the point at which this adjustment took place, we fitted a sigmoidal curve to each individual flight path (Fig. 4c, d). Using this sigmoidal fit, we determined the turning point of the curve, and thus the point at which 50% of the correction manoeuver had been performed, as well as the point at which the sigmoidal curve diverged 10% from its baseline. On average, both measures did not differ between animals flying from a condition of *symmetric* to *asymmetric* optic flow and vice versa (Fig. 4e, f). There was also no significant difference in the average speed before turning (in the first 300 mm of the tunnel). The median of the 10% divergence from baseline was smaller than 0 in both groups indicating that, on average, the hawkmoths responded to the change in translational optic flow before they were parallel to the point of change (Fig. 4f). Nevertheless, the spread in response distance was quite large, and many animals only started to change course once they were level with or had passed the pattern switch.

In the *symmetric* part of the tunnel, the hawkmoths were flying close to the midline but flew closer to the grey wall in the *asymmetric* part of the tunnel. Thus, hawkmoths that responded at the same longitudinal coordinate of the tunnel would perceive the pattern change at a more frontal viewing angle when flying from the *asymmetric* to the *symmetric* condition than those flying in the opposite direction. To account for this, we calculated the perceived viewing angle of each individual hawkmoth (Fig. 4h), given its 10% turn position (Fig. 4f) and their distance to the pattern-switch wall. A viewing angle of 90° denotes a turn point of the hawkmoth exactly lateral of the pattern switch, while angles large than 90° result from turns when the hawkmoth already passed the switch point. The viewing angle was significantly larger when hawkmoths flew from the *asymmetric* to the *symmetric* configuration than vice versa (Fig. 4h).

### Spatial and temporal response cutoff

Based on these measured viewing angles, we estimated the effective spatial frequencies the hawkmoths perceived with each pattern wavelength (Table 1). This analysis revealed that the effective spatial frequency the hawkmoths responded to, given their median viewing angle of 71.4° in the *symmetric* pattern switch configuration, was 0.22 cyc/°, and 0.12 cyc/° in the *asymmetric* configuration (with a median viewing angle of 82.8°).

In addition to the spatial frequencies at response cutoff, we also determined the effective temporal frequency at which hawkmoths perceived the different pattern wavelengths in the tunnel from the average flight speed at each pattern wavelength. Interestingly, the average flight speed in the *asymmetric* configuration was distinctly higher than in the *symmetric* configuration (Figs. 2c, 3c, Fig. S2c). This difference in flight speed also changed the temporal frequencies that the hawkmoths experienced at the respective spatial wavelengths in both configurations: hawkmoths experienced an average of 31 Hz of stripe frequency for the 2.5 cm wavelength stimuli in the *asymmetric* (Fig. S4A) and 41 Hz for the 1.3 cm wavelength in the *symmetric* configuration (Fig. S4B, Table 1).

### The role of body size on flight behaviour

Since the behavioural analysis so far integrated the entire population of hawkmoths across their range of body sizes (Fig. 1d), we further analysed what effect the body size of each individual hawkmoth had on the flight responses in the tunnel—for example, whether large hawkmoths had higher flight speeds than smaller ones. We first analysed the effects of body size on the different flight parameters across spatial wavelengths. To avoid pooling responses from visual conditions that induced stark changes in flight parameters that might obscure potential size-related effects (such as changes in flight speed), we separated the responses in two groups: those from resolvable pattern wavelengths that induced significant translational optic flow responses, and from finer patterns and the control condition for which no significant optic flow responses were observed.

**Fig. 5:**
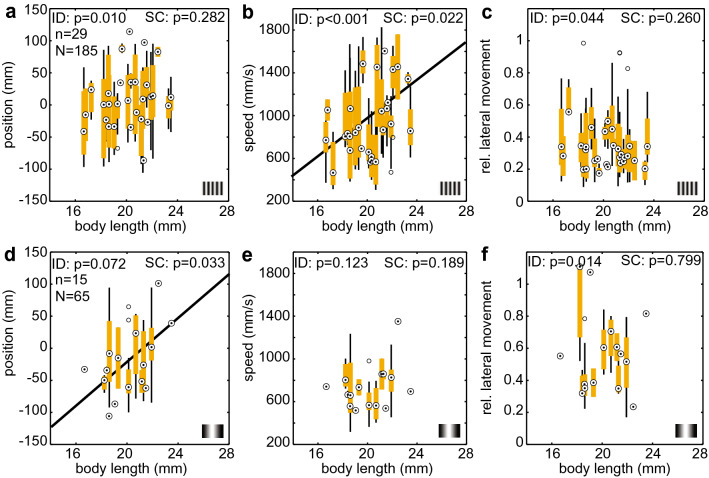
Individual flight behaviour and correlation with body length in the *asymmetric* condition. To assess the flight responses in comparable categories, we split the conditions into **a**–**c** unresolvable (< 2.5 cm) and **d**–**f** resolvable (≥ 2.5 cm) pattern wavelengths. Each boxplot displays the flight parameters of one hawkmoth, based on all flight tracks obtained from this individual in the respective conditions. Plots display the median, the 25th and 75th percentile and the whiskers display the data range excluding outliers. ID (individual differences) represents the statistical result of comparing the flight parameters between individual hawkmoths (Kruskal–Wallis test), and SC (size correlation) shows the results of a linear Pearson correlation analysis (Table S3). Where the linear correlation was significant (*p* < 0.05), the linear regression line is shown in black

For the *asymmetric* stimulus presentation, we used the cutoff wavelength of 2.5 cm, determined on a population level (Fig. 2), to separate the conditions. Across all three flight parameters, we found significant individual effects (Fig. 5a–c, Table S3), suggesting differences in individual flight responses. For the unresolvable gratings, there were individual effects in lateral movement (Fig. 5f) but not in median position or speed (though the number of individuals in both conditions was low and might, therefore, not have allowed for sufficient statistical power in comparison to the other patterns). We also found a statistically significant correlation between body size and average speed for the unresolvable patterns (Fig. 5b), indicating that larger hawkmoths flew faster than smaller ones in the absence of strong translational optic flow cues. Furthermore, for the resolvable patterns (Fig. 5d), we found a significant correlation between body size and the median flight position of the hawkmoths, suggesting that larger hawkmoths flew at a greater distance from the patterned wall than smaller ones.

To assess the effects of body size (separated into *large* and *small* animals, with the median body size of 20.52 mm separating the two groups), given the pattern condition (*resolvable*, *unresolvable*) and the individual identity of each hawkmoth, we fitted linear mixed-effects models to the data. In the *asymmetric* configuration, for median tunnel position, flight speed and lateral movement, the model with fixed effects was a significantly better fit than the null model that accounted only for individual differences (AIC, d vs d, likelihood ratio test, median position: *Χ*^2^ = 8.83, df = 3, *p* = 0.031, flight speed: *Χ*^2^ = 18.54, df = 3, *p* < 0.001, lateral movement: *Χ*^2^ = 38.78, df = 3, *p* < 0.001). Moreover, there was no significant interaction between pattern condition and animal body size for both flight parameters (AIC, d vs d, likelihood ratio test, median position: *Χ*^2^ = 2.76, df = 1, *p* = 0.097 flight speed: *Χ*^2^ = 0.23, df = 1, *p* = 0.632, lateral movement: *Χ*^2^ = 0.15, df = 1, *p* = 0.70). In line with our population-level data, the median position was significantly shifted towards the grey tunnel side in the *resolvable* condition (post-hoc test with Tukey contrasts, *z* = − 2.80, *p* = 0.005), flight speeds were significantly lower (*z* = -2.71, *p* = 0.007), while lateral movement was significantly larger (*z* = 4.83, *p* < 0.001). For median position and lateral movement, there was no significant difference between *large* and *small* body sizes (median position: *z* = 0.27, *p* = 0.787, lateral movement: *z* = − 0.327, *p* = 0.744). Body size did have a significant effect on flight speed (*z* = 2.57, *p* = 0.010), with larger animals faster than small ones, confirming the effect visible in Fig. 5b.

**Fig. 6:**
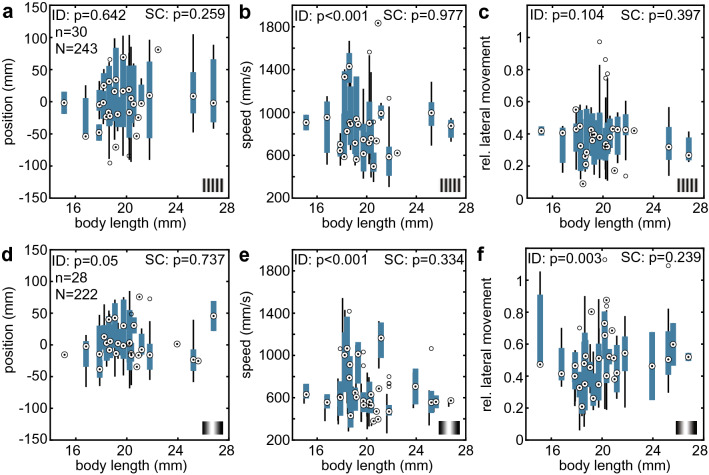
Individual flight behaviour and correlation with body length in the *symmetric* condition. To assess the flight responses in comparable categories, we split the conditions into **a**–**c** unresolvable (< 1.3 cm) and **d**–**f** resolvable (≥ 1.3 cm) pattern wavelengths. Each boxplot displays the flight parameters of one hawkmoth, based on all flight tracks obtained from this individual in the respective conditions. Plots display the median, the 25th and 75th percentile and the whiskers display the data range excluding outliers. ID (individual differences) represents the statistical result of comparing the flight parameters between individual hawkmoths (Kruskal–Wallis test), and SC (size correlation) shows the results of a linear Pearson correlation analysis (Table S4)

In the *symmetric* condition, we used a pattern wavelength of 1.3 cm as the threshold for the resolvable pattern category (Fig. 3). For the unresolvable patterns, individual differences were only observed in flight speed (Fig. 6b, Table S4). With the resolvable patterns, we observed individual differences in all three flight parameters (Fig. 6d–f, though the differences in position were only marginally significant). We did not observe a correlation between body length and any of the three flight parameters across pattern types.

**Fig. 7:**
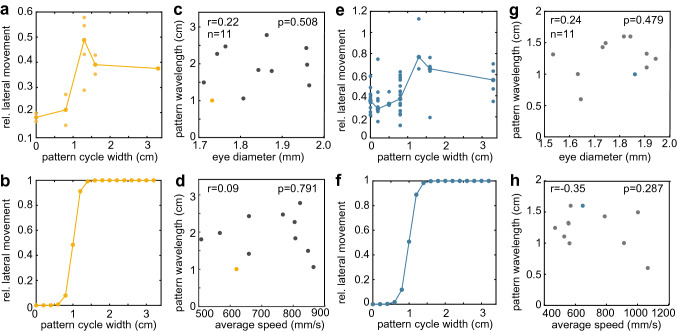
Effect of body length on the spatial resolution in the *asymmetric* and *symmetric* configurations. **a**, **e**, **b**, **f** The relative lateral movement of individual hawkmoths at each pattern wavelength (*asymmetric*: **a**, *symmetric*: **e**) was used to fit a sigmoidal function (*asymmetric*: **b**, *symmetric*: **f**), to describe the change in flight behaviour with pattern wavelengths. The 50% turning point of the function was used as a measure for the response cutoff with respect to the spatial frequency of the presented patterns. **c**, **g**, **d**, **h** These response cutoffs were set in relation to the individuals’ average eye diameter (*asymmetric*: **c**, *symmetric*: **g**), but no significant linear correlation was found (Pearson correlation). There was also no linear correlation with the cutoff pattern wavelength and the individuals’ average speed (*asymmetric*: **d**, *symmetric*: **h**)

As in the *asymmetric* configuration, in the *symmetric* configuration, the linear model with fixed effects provided a significantly better fit than the null model accounting only for individual differences for all three flight parameters (AIC, d vs d, likelihood ratio test, median position: *Χ*^2^ = 11.76, df = 3, *p* = 0.008, flight speed: *Χ*^2^ = 96.4, df = 3, *p* < 0.001, lateral movement: *Χ*^2^ = 49.29, df = 3, *p* < 0.001). There was no significant interaction between pattern condition and animal body size for both flight parameters (AIC, d vs d, likelihood ratio test, median position: *Χ*^2^ = 1.88, df = 1, *p* = 0.170, flight speed: *Χ*^2^ = 0.44, df = 1, *p* = 0.50, lateral movement: *Χ*^2^ = 1.65, df = 1, *p* = 0.20). In line with the population data, flight speeds were significantly lower in the *resolvable* condition (post-hoc test with Tukey contrasts, *z* = − 6.50, *p* < 0.001), while lateral movement was significantly larger (*z* = 3.85, *p* < 0.001) and median position did not differ (*z* = 0.71, *p* = 0.477). For all flight parameters, there was no significant difference between *large* and *small* body sizes (the median body size of 20.28 mm separating the two groups, median position: *z* = 1.00, *p* = 0.316, flight speed: *z* = − 0.72, *p* = 0.475, lateral movement: *z* = 0.45, *p* = 0.651).

### Correlation between eye size and spatial response cutoffs

To test if there was a correlation between the individual hawkmoths’ eye size and their spatial acuity, we analysed each individual hawkmoth’s flight responses in the tunnel. We focused on the lateral movement parameter, because it provided a clear response readout in both the *asymmetric* and the *symmetric* configuration (Fig. 7a example *asymmetric*, E *symmetric*). We fitted a sigmoidal curve to the lateral movement of an individual hawkmoth’s flight tracks across all pattern wavelengths (Fig. 7b, f). From this curve, we extracted the spatial wavelength at the turning point as a measure for the response cutoff. There was no significant linear correlation between the average eye diameter of each individual and their spatial response cutoff in either the *asymmetric* or in the *symmetric* configuration (Fig. 7c, g). There was also no significant linear correlation between the response cutoff and either the individuals’ flight speed (Fig. 7d, h), or their body size (Fig. S3A, E).There was also no significant correlation between the perceived spatial frequency (calculated from each hawkmoths’ median position in the tunnel) and the individuals’ eye diameter (Fig. S3C, G), body length (Fig. S3B, F), or average flight speed (Fig. S3D, H).

## Discussion

In this study, we investigated the spatial response cutoffs of flight control behaviour in an insect with superposition compound eyes. Unlike previous studies in other insects, we found different spatial cutoffs to optic flow-based flight responses depending on the configuration in which the optic flow was presented. While we did observe that flight characteristics differed significantly between individuals, this was not related to their body size, and we also did not observe a correlation between body size and spatial acuity.

### Hawkmoth responses to translational optic flow compared to other insects

On a general level, the hawkmoths’ responses to the lateral patterns in the tunnel were similar to those previously described in hawkmoths (Stöckl and Kelber 2019) and other insects (Kirchner and Srinivasan 1989; Baird et al. 2010; Dyhr and Higgins 2010; Kern et al. 2012; Chakravarthi et al. 2018). Their centring response and avoidance of higher translational optic flow regions is consistent with a general strategy of adjusting their lateral position in the tunnel to balance the perceived translational optic flow in both eyes (Srinivasan et al. 1999). While the hawkmoths’ responses to resolvable translational optic flow patterns were similar across pattern wavelengths in the *asymmetric* configuration and for flight speed and lateral movement in the *symmetric* configuration, the centring response differed in strength depending on pattern wavelength (Fig. 3). It was strongest for 1.6 cm (0.176 cyc/° in the fronto-lateral visual field), and weaker for smaller and larger spatial wavelengths.

In addition to their position control and the reduction in flight speed upon reception of translational optic flow in the *symmetric* configuration, we also noted an increase in lateral movement (that is movement perpendicular to the direction of travel along the tunnel). This reliably correlated with the change in the other parameters that occurred when the hawkmoths could perceive the grating pattern. At first, it might seem counterintuitive that the flight paths of animals perceiving translational optic flow should have more lateral contributions than animals that did not, as translational optic flow is generally thought to help insects stabilise their course, and thus support straighter flight paths with lower lateral components than in featureless environments (Linander et al. 2017). And indeed, the overall lateral drift of the hawkmoths was low for resolvable patterns, as indicated by the concentration of their median position around the midline of the tunnel (Fig. 3). In line with this, the overall variance in position of the flight tracks did not significantly increase in the translational optic flow conditions (Fig. S2A). Thus, the increased lateral movement did not represent an impaired course control, but instead was caused by small oscillations around relatively straight flight tracks.

These lateral oscillations might either represent artefacts caused by the highly contrasting single-wavelength patterns used in the flight tunnel, which might have induced oscillations in response to the repetitive stripes on the tunnel walls. If this were the case, these oscillations should have correlated with the pattern wavelength, with finer resolvable stripes generating proportionally more lateral movement, which was not the case (Fig. 3d). Recent findings in honeybees suggest that regular lateral oscillations might be used to gage the distance to the ground and thus regulate flight height (Baird et al. 2021). However, this strategy works optimally when the lateral oscillations are generated independent of the perceived visual texture—which was not the case in our study, where hawkmoths generated significantly stronger lateral movements for patterns generating resolvable translational optic flow than for those that did not. It therefore remains to be investigated in the future studies whether hawkmoths use these lateral movements to gage their height above the ground, albeit with a potentially altered strategy from honeybees. A further explanation for the occurrence of lateral movements could be the particular flight mode of the hawkmoths when crossing the tunnel, because we noticed a strong log-linear correlation between lateral movement and flight speed (Fig. S2B). This suggests that the lateral oscillations might be a side effect of slow forward flight in hawkmoths—a hypothesis that remains to be tested.

Our results show that hawkmoths generally responded to a change in translational optic flow before they were parallel to the actual switch in pattern, though the spread between individuals was large. This suggests that hawkmoths respond to changes in translational optic flow cues in their fronto-lateral visual field. Interestingly, we did not observe a difference in response position for hawkmoths flying either into symmetric cues (stronger translational optic flow cues) from an asymmetric presentation (weaker cues), or the other way around. This differs from results in bumblebees, which respond earlier to a switch from weak to strong translational optic flow cues than vice versa (Linander et al. 2015). This strategy seems adaptive, as the threat to collide with potential obstacles sensed through an increase in translational optic flow is higher when flying from a strong to weak translational optic flow scenario. An earlier steering response would remedy this threat. On the other hand, the collision threat is low when animals move into a part of the tunnel with lower translational optic flow, and correspondingly, bumblebees responded to the change in optic flow only when they passed the pattern change. It was rather surprising that the hawkmoths did not show a similar change in strategy. It is possible that this is related to their agile flight skills and their ability to hover and stop in mid-air. Their tolerance to coming close to obstacles might, therefore, be higher than that of bumblebees, and a change in strategy might only be observed in narrower tunnels or with even stronger optic flow cues.

### Spatial response cutoffs depend on the optic flow configuration

Somewhat unexpectedly, we observed a difference in spatial response cutoffs between the *asymmetric* and the *symmetric* configuration. In the *symmetric* configuration, the spatial cutoff frequency was 0.22 cyc/° at the determined fronto-lateral viewing angle of 71.4°, which is consistent with the 50% cutoff frequency described for the hummingbird hawkmoths’ wide-field motion neurons of 0.23 cyc/° at comparable light intensities (Stöckl, O’Carroll and Warrant 2017). In contrast, in the *asymmetric* condition, the spatial cutoff of optic flow-based responses was 0.11 cyc/°—lower than that of motion neurons at four orders of magnitude lower light intensity (Stöckl et al. 2017c). One big difference between the *symmetric* and the *asymmetric* configuration was that the flight speed of the animals (Figs. 2C, 3c) was about 75% lower in the *symmetric* configuration for resolvable spatial frequencies (Fig. S2C). This difference likely resulted from the different magnitudes of optic flow the hawkmoths perceived across their entire visual field in these two scenarios, and suggests that they determine their speed by integrating translational optic flow over both eyes, as bumblebees do (Linander et al. 2015). The difference in flight speed also affected the temporal frequencies the hawkmoths experienced at the respective spatial wavelengths in both configurations: at 31 Hz in the *asymmetric* and 41 Hz in the *symmetric* configuration, these were distinctly higher than the 12 Hz cutoff recorded in wide-field motion neurons at comparable light intensities (Stöckl et al. 2017c). It is very likely that the cutoffs, measured in the neurons of restrained hawkmoths, distinctly underrepresent the temporal tuning in actively flying insects, since octopamine induced state-depended changes in homologues of these neurons in flies have been shown to shift the temporal optimum to higher frequencies (Chiappe et al. 2010; Jung et al. 2011). In flies, the change from quiescent to flight increased the temporal response cutoff more than twofold (Jung et al. 2011). If this was the case in hawkmoths too, the observed behavioural response cutoffs might be explained by a shift in temporal tuning of their wide-field motion neurons—and the fact that behavioural responses are likely possible at lower than 50% optimal response strength of the motion neurons.

### Temporal resolution as the limiting parameter in translational optic flow responses

Given the spatial and temporal frequencies at which the hawkmoths could not resolve the patterns in the tunnel any longer, it is conceivable that the limiting factor in this configuration was their temporal response optima not their spatial tuning. At 31 Hz and 41 Hz, the temporal response cutoffs differ less than the twofold difference in spatial cutoff between the configurations. Moreover, the temporal frequency that hawkmoths would have experienced at the pattern wavelengths that were finer than their cutoffs (1.6 cm in the *asymmetric*, and 0.8 cm in the *symmetric* configuration) are larger (at 52 Hz and 112 Hz, respectively) than those received in any configuration in which hawkmoths still responded to the patterns (41 Hz). Thus, the resulting cutoff responses in both configurations would be consistent with an upper temporal resolution limit of ca. 40 Hz, while a spatial resolution limit does not explain the different response limits we found between the *asymmetric* and *symmetric* conditions.

It is inherent in the free-flight study design that it is not possible to resolve whether the behavioural response cutoffs are based on spatial or temporal resolution limits. However, previous spatial cutoffs determined in bumblebees (Dyhr and Higgins 2010; Chakravarthi et al. 2017) were consistent with the optical limits of the bees’ eyes and their expected spatial resolution (Spaethe 2003; Taylor et al. 2019). Our data provides the first evidence for a temporal, rather than a spatial limit of an insects’ translational optic flow responses to fine spatial details. This is consistent with the fact that the temporal tuning of hawkmoth motion neurons is distinctly lower than that of bumblebees (O’Carroll et al. 1996, 1997; Stöckl et al. 2017a, b), likely as a result of the hummingbird hawkmoths’ nocturnal ancestry, and the need to resolve lower temporal frequencies during hovering flight. Nevertheless, the hawkmoths crossed tunnels of the same proportions at similar speeds as bumblebees (Linander et al. 2015; Chakravarthi et al. 2017, 2018). So they would have experienced similar temporal frequencies at the same spatial wavelengths. Thus, given the same conditions, hawkmoths are limited by their temporal tuning at much lower spatial wavelengths than bumblebees, explaining why we saw these effects in our experiments that were not apparent in previous bumblebee work.

It is interesting to note that if this interpretation of the data holds, hawkmoths did not adapt their flight speed to optimise their optic flow perception. While they cannot alter their spatial resolution limits, by flying slower, they could increase the range of resolvable spatial frequencies that might be cutoff by their temporal resolution. However, our data offers no indication that the hawkmoths flew slower when presented with finer patterns. It is possible that they already reached their minimum forward flight speed at higher wavelengths and had no physical room for adjustments. In addition, previous results have shown that hummingbird hawkmoths weigh optic flow information for flight control stronger in their ventral visual field than in their lateral one—even if cues inducing translational optic flow are present in the lateral view of both eyes (Bigge et al. 2021). Since the floor of our flight tunnel likely generated only weak translational optic flow, hawkmoths might have retained their relatively high flight speeds, despite them being mal-adapted for resolving the patterns presented laterally in the tunnel. Moreover, a flight speed adjustment in response to the spatial structure of the surrounding visual scene might simply not be part of the hawkmoths’ neural and behavioural repertoire, because the spatial structure of their natural environment generally contains contrasts at a range of spatial frequencies (van der Schaaf and van Hateren 1996; Stöckl et al. 2017a, b), which sufficiently stimulate their optic flow system at most flight speeds.

### Flight performance does not correlate with body size

As in a previous study (Stöckl et al. 2019), we found strong individual variation in the flight parameters between hawkmoths in both the *symmetric* and *asymmetric* configurations (Figs. 5, 6). While some individual hawkmoths had very little variation in their median tunnel position for consecutive flights, indicating that they took consistent paths through the tunnel, most individuals had a wide variation in median position, lateral movement and speed across consecutive flights, indicating that most hawkmoths do not seem to learn a specific path through the tunnel in contrast to bumblebees and honeybees (Serres et al. 2008; Bertrand et al. 2021). Except for one instance, the individual differences in flight parameters between hawkmoths were not correlated with body size (Figs. 5, 6). While one might not expect a connection between body size and lateral movement or median flight position, a correlation between body size (and wing span) with flight speed could be expected (Henningsson and Bomphrey 2013). Such a correlation should only manifest in conditions where the hawkmoths did not reduce their flight speed due to the perceived translational optic flow, as wing span likely only influenced the hawkmoths’ maximum flight speeds. Indeed, the one significant correlation of flight parameters with body size was the flight speed with non-resolvable patterns in the *asymmetric* condition. Larger animals here flew faster, as would be expected if wing span was influencing flight speed in the tunnel. However, we did not observe a similar correlation in the non-resolvable symmetric conditions. Thus, if there was a connection between body size and flight speed in the tunnel, it was not dominant over the individual variation in hawkmoth flight strategies.

### Spatial resolution does not scale with eye size

In our study, we did not observe a correlation between the eye size of the hawkmoths, and their spatial response cutoffs (Fig. 7, Fig. S3). This could be an indication that spatial acuity is not sacrificed to retain contrast sensitivity in the high sensitivity superposition compound eyes to the same degree as it is in apposition compound eyes of other insects. The lack of correlation between eye size and spatial cutoff is not for a lack in range of eye sizes tested: compared to fruit flies, where a small but significant difference in spatial cutoff was found between individuals differing by 33% in eye area—corresponding to approximately 14% in eye diameter (Currea et al. 2018), the range of eye sizes in our study was twice as large. Moreover, the number of observations and range of body and eye sizes in the present study was comparable to Spaethe (2003), who found that the eye size of *Bombus terrestris* scaled significantly with spatial acuity in a target detection task—although the effect observed in bumblebees was likely not due to a limit of spatial acuity, but contrast sensitivity of individual ommatidia. Despite there being a significant correlation between eye size and spatial acuity in the apposition compound eyes of bumblebees (Spaethe 2003; Taylor et al. 2019), an optic flow-based flight control task in bumblebees did not reveal a correlation between the spatial cutoffs and body size (Dyhr and Higgins 2010), nor did a wide-field spatial discrimination task (Chakravarthi et al. 2016). One possible explanation for why the scaling relationship of spatial resolution does not manifest in such free-flight behavioural tasks is that the optic flow the insects perceive is limited by both the spatial and temporal resolution. And while those can be precisely controlled in open-loop experiments with fixed insects, in free-flight paradigms, varying the spatial wavelength of the stimuli also varies the temporal frequencies the insects perceive—depending on their flight speeds. Our results suggest that it might indeed have been the temporal, rather than the spatial response cutoffs that were limiting the hawkmoths’ optic flow responses. Nevertheless, in the *symmetric* configuration, hawkmoths did approach the spatial 50% cutoff frequency of their wide-field motion-sensitive neurons (Stöckl et al. 2017c), thus making it possible that spatial resolution limits could have influenced the responses in this configuration, particularly in smaller hawkmoths. Whether this was the case in our or previous experiments remains to be tested with either tethered-flight [such as (Currea et al. 2018)] or free flight in virtual reality [such as (Fry et al. 2009)]. The second possibility for a lack of correlation between spatial response cutoffs and eye size in hawkmoth and bumblebee wide-field movement tasks is that smaller eyes are optimised for higher spatial acuity, at the cost of contrast sensitivity—in line with the observations of (Spaethe 2003). Differences in contrast sensitivity might not manifest as clearly in wide-field tasks than in small-target detection tasks, since the visual system of the insect can increase its sensitivity by pooling responses over a large visual area. This hypothesis would fit well with the hawkmoths’ superposition compound eye design, which provides higher single-ommatidia sensitivity than apposition eyes of similar size (Stöckl et al. 2017c), thus potentially reducing the need to sacrifice resolution to retain sufficient sensitivity in smaller eyes. Future anatomical studies of the allometric scaling in hawkmoth eyes, combined with studies of contrast sensitivity of hawkmoth flight control will reveal whether the effect of larger bodies and eyes manifests in contrast sensitivity, rather than spatial acuity in this insect group.

## Conclusion

Taken together, hummingbird hawkmoths show different spatial frequency cutoffs of their centring responses with sinusoidal gratings, depending on whether they are presented on one side or on both sides of a flight tunnel. Their flight behaviour revealed high individual variability, but we did not find any correlation in response cut-off with body or eye size. The difference in response cutoffs was consistent with a temporal, rather than a spatial limit, to the hawkmoths’ centring behaviour. What consequences this limitation might have for their natural flight behaviour, when a wider range of spatial frequencies is available to their visual system and flight speeds are higher, remains an open question for further investigation.

## Supplementary Information

Below is the link to the electronic supplementary material.

Supplementary file1 (PDF 2196 kb)

## Declarations

### Data availability

Data supporting the presented results are available at Figshare (https://figshare.com/s/d66d0fe7adc00bd73d3d).

### Code availability

The custom-written MATLAB-code used to analyse the data and prepare the figures is available from the corresponding author upon request.
