# Supplementary material for: Spatial tuning of translational optic flow responses in hawkmoths of varying body size
Source: J Comp Physiol A Neuroethol Sens Neural Behav Physiol. 2021 Dec 10;208(2):279–96. doi: 10.1007/s00359-021-01530-1 (PMC8934765; doi:10.1007/s00359-021-01530-1)
Supplement: Supplementary file 1 — Supplementary file1 (PDF 2196 kb) [file 359_2021_1530_MOESM1_ESM.pdf]

## Supplementary Figures

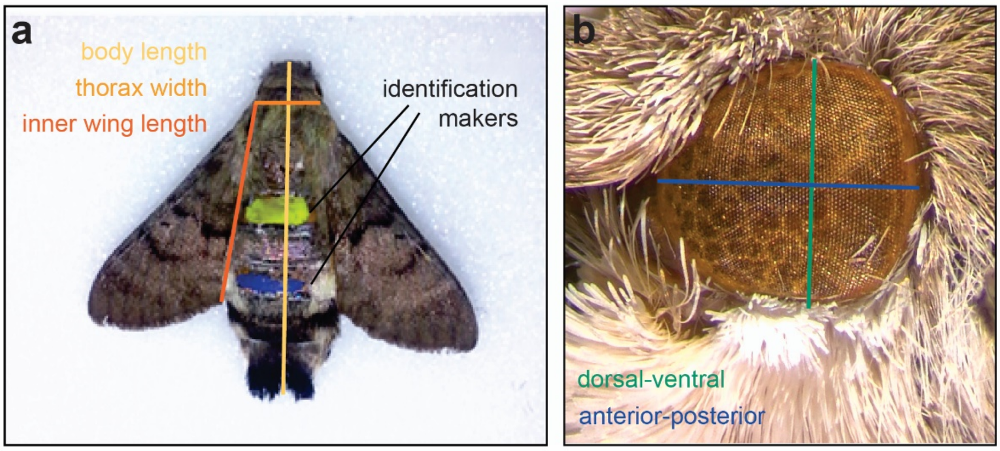


Figure S1. **Hawkmoth body and eye measurements**. **A** Individually marked hawkmoth. Each moth in the population experiments (*asymmetric* and *symmetric* set-up) was marked at the lower and upper abdomen with a specific colour code. Anatomical measurements of the hawkmoth size were performed after experiments from photographs of the moths as indicated. **B** Measurements of the eyes' diameter in the dorsal-ventral and anterior-posterior axis were performed using photographs taken with a stereomicroscope as indicated.

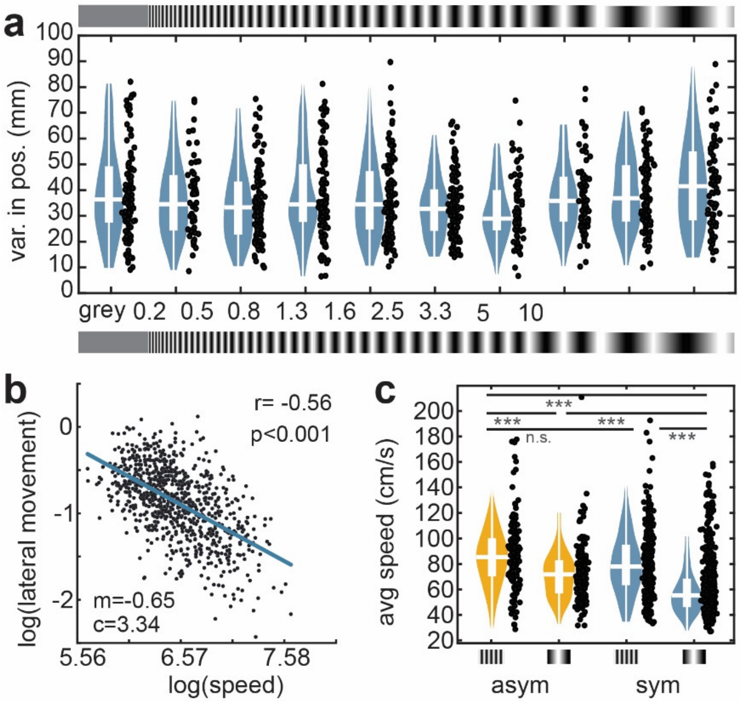


Figure S2: **In flight variation and flight speed of the hawkmoths in the *symmetric* configuration.** **A** Variation in lateral position within each flight track in each condition. No significant difference between the different spatial conditions and the grey condition was found (Kruskal-Wallis-test). Boxplots (white) within the violin plots display the median, the 25th and 75th percentile and the

whiskers denote the data range excluding outliers (values extending more than 1.5 interquartile ranges from the upper and lower box limits). **B** Correlation between the log-transformed flight speed and relative lateral movement of all flight tracks across spatial wavelength conditions in the *symmetric* configuration. The strength of the linear correlation coefficient of the log-transformed data is given by r, and the statistical significance of the Pearson correlation coefficient by p. The slope of the linear model is given by m, the intercept by c.

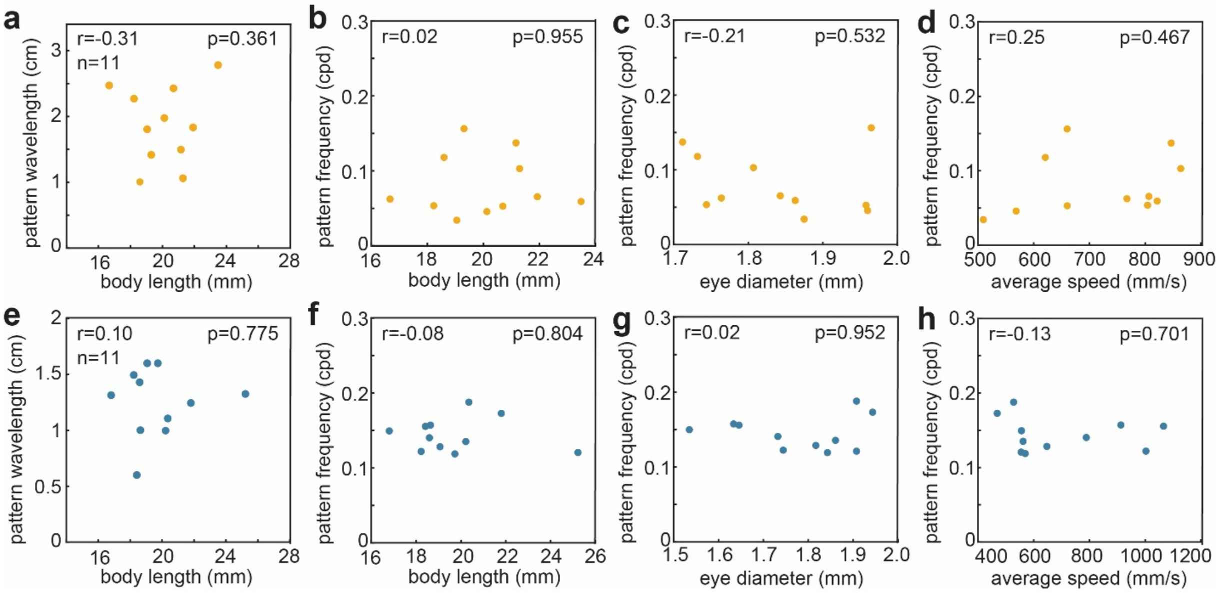


Figure S3: **Effect of body length and eye diameter on the spatial resolution in the *asymmetric* and *symmetric* configuration**. **A,E** The spatial response cut-offs in relation to the individuals' body length (*asymmetric*: **A**, *symmetric*: **E**). **B-D**,**F-G** The spatial response cut-offs calculated as perceived spatial frequency based on each individuals' median position in the tunnel, in relation to the individuals' body length (*asymmetric*: **A**, *symmetric*: **E**), eye diameter (*asymmetric*: **C**, *symmetric*: **G**), and average speed (*asymmetric*: **D**, *symmetric*: **H**).

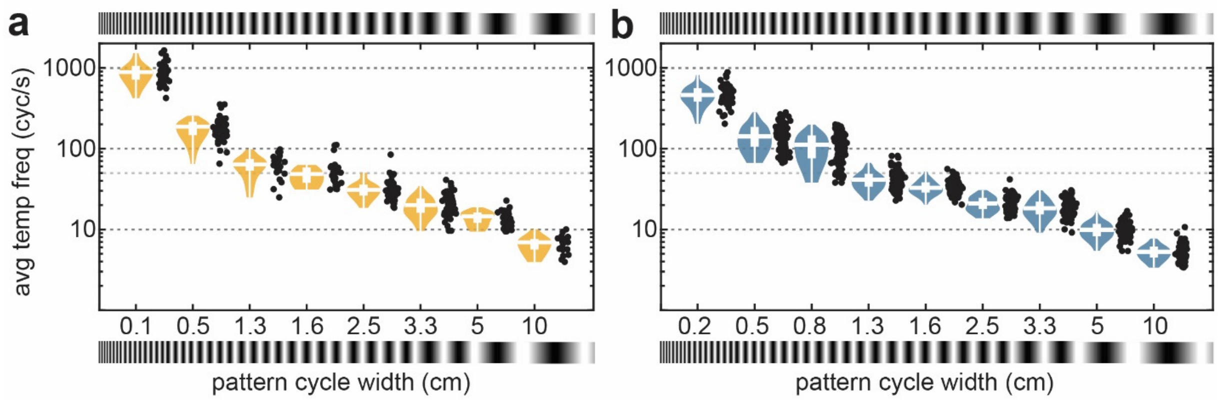


Figure S4: **Perceived temporal frequency of the hawkmoths in the *asymmetric* and *symmetric* configurations.** The perceived temporal frequency was calculated for each pattern wavelength

based on each flightpaths' average speed in the **A** asymmetric and **B** symmetric configuration. The number of trajectories N for each pattern condition shown in **A** was: 0.1 N=40, 0.5 N=46, 1.3 N=57, 1.6 N=68, 2.5 N=53, 3.3 N=76, 5 N=44, 10 N=23 and in B was: 0.2 N=59, 0.5 N=107, 0.8 N=114, 1.3 N=111, 1.6 N=115, 2.5 N=78, 3.3 N=69, 5 N=99, 10 N=81. Boxplots (white) within the violin plots display the median, the 25th and 75th percentile and the whiskers denote the data range excluding outliers (values extending more than 1.5 interquartile ranges from the upper and lower box limits).

### ***Statistical results***

Table S1. Statistical results of flight parameters in the *asymmetric* configuration. The type of data and statistical tests used are indicated in the respective rows, and the number of analysed flights is given for each comparison.

| ***asymmetric configuration*** | | | | | | | | | |
|---|---|---|---|---|---|---|---|---|---|
| ***wavelength (cm)*** | grey | 0.1 | 0.5 | 1.3 | 1.6 | 2.5 | 3.3 | 5 | 10 |
| ***number of flights*** | *37* | *38* | *44* | *57* | *68* | *51* | *76* | *44* | *21* |
| *Median lateral position compared to the control (grey) - condition*<br>*ANOVA, df = 8, F = 9.1* | | | | | | | | | |
| ***post-hoc test (p value)*** | | *1.000* | *0.899* | *0.998* | *0.950* | *0.052* | *<0.001* | *<0.001* | *0.009* |
| *Median lateral position compared to the midline*<br>*Mann-Whitney-U-test* | | | | | | | | | |
| ***(p value)*** | *0.446* | *0.913* | *0.197* | *0.706* | *0.296* | *<0.001* | *<0.001* | *<0.001* | *0.003* |
| *Average speed compared to the control (grey) - condition*<br>*Kruskal-Wallis-Test, df = 8, $X^2$ = 58.3* | | | | | | | | | |
| ***post-hoc test (p value)*** | | *1.000* | *0.999* | *1.000* | *1.000* | *0.893* | *0.003* | *0.041* | *0.022* |
| *Lateral movement compared to the control (grey) – condition*<br>*Kruskal-Wallis-Test, df = 8, $X^2$ = 58.9* | | | | | | | | | |
| ***post-hoc test (p value)*** | | *1.000* | *1.000* | *1.000* | *0.999* | *0.009* | *<0.001* | *<0.001* | *0.108* |

Table S2. Statistical results of flight parameters in the *symmetric* configuration. The type of data and statistical tests used are indicated in the respective rows, and the number of analysed flights is given for each comparison.

| ***symmetric configuration*** | | | | | | | | | |
|---|---|---|---|---|---|---|---|---|---|
| ***wavelength (cm)*** | grey | 0.2 | 0.5 | 0.8 | 1.3 | 1.6 | 2.5 | 3.3 | 5 |
| ***number of flights*** | *97* | *58* | *106* | *113* | *111* | *115* | *77* | *69* | *99* |
| *Variance in median lateral position compared to the control (grey) - condition Brown–Forsythe test* | | | | | | | | | |
| ***post-hoc test (p value)*** | | 0.343 | 0.612 | 0.315 | 0.001 | 0.001 | 0.001 | 0.014 | 0.004 |
| ***test statistic*** | | 0.906 | 0.259 | 1.017 | 20.067 | 54.773 | 35.648 | 6.194 | 8.622 |
| *Average speed compared to the control (grey) - condition Kruskal-Wallis-Test, df = 9, $X^2$ = 308.1* | | | | | | | | | |
| ***post-hoc test (p value)*** | | 0.327 | 0.450 | 1.000 | 0.001 | 0.001 | 0.001 | 0.001 | 0.001 |
| *Lateral movement compared to the control (grey) – condition Kruskal-Wallis-Test, df = 9 $X^2$ = 157.3* | | | | | | | | | |
| ***post-hoc test (p value)*** | | 0.296 | 0.989 | 0.988 | 0.019 | 0.001 | 0.001 | 0.001 | 0.001 |

Table S3: Statistical results of the effect of individual identity on the flight parameters median position, average speed and lateral movement in the *asymmetric* configuration (Kruskal-Wallis-test (KW) with individual identity as group factor). Flight tracks from different spatial conditions were grouped as indicated. The flight parameters were further tested for a linear correlation with body length using a Pearson-Correlation-test (PC). ID: Individual effects, SC: size correlation. Data presented in Fig. 5. Individual animals used in this analysis: n=44.

| ***asymmetric configuration*** | **Type of test** | **Median position** | **Average speed** | **Lateral movement** |
|---|---|---|---|---|
| ***Pattern wavelengths <2.5cm*** | ID: KW | *p = 0.010* | *p< 0.001* | *p = 0.044* |
| | SC: PC | *p = 0.282* | *p = 0.022* | *p = 0.260* |
| | | R = 0.21 | R = 0.43 | R = -0.22 |
| ***Pattern wavelengths >2.5cm*** | ID: KW | *p = 0.072* | *p = 0.012* | *p = 0.014* |
| | SC: PC | *p = 0.033* | *p = 0.189* | *p = 0.799* |
| | | R = 0.55 | R = 0.36 | R = -0.07 |

Table S4: Statistical results of the effect of individual identity on the flight parameters median position, average speed and lateral movement in the *symmetric* configuration (Kruskal-Wallis-test (KW) with individual identity as group factor). Flight tracks from different spatial conditions were grouped as indicated. The flight parameters were further tested for a linear correlation with body length using a Pearson-Correlation-test (PC). ID: Individual effects, SC: size correlation. Data presented in Fig. 6. Individual animals used in this analysis: n=34.

| **symmetric configuration** | **Type of test** | **Median position** | **Average speed** | **Lateral movement** |
|---|---|---|---|---|
| ***Pattern wavelengths <1.3cm*** | ID: KW | *p = 0.642* | *p< 0.001* | *p = 0.141* |
| | SC: PC | *p = 0.259* | *p = 0.977* | *P = 0.397* |
| | | R = 0.23 | R = -0.01 | R = -0.17 |
| ***Pattern wavelengths >1.3cm*** | ID: KW | *p = 0.005* | *p< 0.001* | *p = 0.003* |
| | SC: PC | *p = 0.737* | *p = 0.335* | *p = 0.239* |
| | | R = 0.07 | R = -0.19 | R = 0.23 |
